# Non-Pharmacological Interventions for the Hallucinations in Patients with Dementia. A Cross-Over Randomized Controlled Trial

**Published:** 2022-01-31

**Authors:** Dimitriou Tatiana-Danai, Papatriantafyllou John, Konsta Anastasia, Kazis Dimitrios, Athanasiadis Loukas, Ioannidis Panagiotis, Koutsouraki Efrosini, Tegos Thomas, Tsolaki Magda

**Affiliations:** 1 1^st^ Department of Neurology, Aristotle University of Thessaloniki, Greece; 2 2^nd^ Neurology Department, University of Athens, ‘Attikon’ Hospital, Greece; 3 1^st^ Department of Psychiatry, Aristotle University of Thessaloniki, Greece

**Keywords:** Dementia, BPSD, Non-pharmacological, Crossover randomized trial, Hallucinations

## Abstract

**OBJECTIVE:**

Hallucinations is a core characteristic symptom in Lewy Body Dementia (DLB) and Parkinson’s Dementia (PDD). It may also appear at the late stages of Alzheimer’s disease (AD). They are not easily managed, and they are associated with cognitive decline, earlier institutionalization, increased mortality, and increased caregivers’ burden.

**DESIGN/SETTING:**

This is a cross-over RCT. The participants were randomly assigned in 6 different groups of 10 patients each.

**PARTICIPANTS:**

60 dementia patients (different types and stages of dementia).

**INTERVENTIONS:**

The three non-pharmacological interventions used are: A) Validation therapy (VT) in a Psycho-educational program, B) Reminiscence therapy (RT) and C) Music therapy (MT). Each intervention lasted for 5 days and there was an interval of two days, as a wash-out period (all the interventions had a duration of 3 weeks).

**MEASUREMENTS:**

The measurements which were used at baseline were: MMSE, ACE-R, GDS, FRSDD and NPI questionnaire at baseline and after each intervention.

**RESULTS:**

The most effective combination for the reduction of the hallucinations is: VT/Psycho-educational program (p = 0.005), MT (p = 0.007) and RT (p = 0.022). The same combination applies for the caregivers’ distress: VT/Psychoeducational program (p = 0.010) - MT (p = 0.023) - RT (p = 0.036).

**CONCLUSION:**

VT/Psycho-educational program followed by MT, followed by RT is an effective combination of non-pharmacological interventions that can reduce hallucinations in patients with dementia and caregivers’ burden. Non-pharmacological interventions should be further examined as an effective alternative for the reduction of the hallucinations in dementia.

## INTRODUCTION

Hallucinations occur in several types of dementias including Alzheimer’s Disease (AD), Parkinson’s disease dementia (PDD), Lewy Body dementia (DLB) and Frontotemporal Dementia (FTD) [1]. Patients with dementia (PwD) in general experience mostly visual hallucinations. These are defined as “experiencing a visual perception in the absence of an external stimulus”, which means that the patient sees something that others cannot see [1]. There are other types of hallucinations such as auditory, olfactory, and tactile, as well, which also mean that the patient listens or smells or touches things that others do not [2,3]. However, visual hallucinations are the most common. The prevalence estimates from 12%-53% in AD, 45%-65% in DLB, and 25%-40% in PDD patients [4]. No official results have been found for the FTD patients, maybe because of the fact that although FTD causes significant behavior changes, however hallucinations are rare [5]. Their aetiology it is not clear yet, although DLB patients have reduced occipital and parietal perfusion and glucose metabolism [6]. Visual hallucinations are included as a core criterion for the diagnosis of DLB. They are associated with cognitive decline, earlier institutionalization, increased mortality, and caregivers’ burden [7].

Atypical antipsychotics were studied for the management of the hallucinations in PwD [8]. However, their side effects are severe: the antipsychotic agents can worsen extrapyramidal symptoms and the cognitive abilities [8]. Cholinesterase inhibitors may help visual hallucinations [9]. Rivastigmine is well tolerated by PwD when the dose escalates slowly [9]. Primavanserin has some positive effects however, treatments need to be carefully examined and reviewed often [10]. On the other hand, the non-pharmacological interventions have no side effects, are pleasurable for the PwD and the caregivers, and can reduce effectively some behavioral and psychological problems in dementia (BPSD) [11].

The aim of the current study is to examine if there is a combination of non-pharmacological interventions that can effectively reduce the hallucinations in PwD and reduce the caregivers’ burden, as well.

## METHOD AND MEASUREMENTS

### Subjects

Sixty patients (n = 60) with a diagnosis of dementia and hallucinations were included. The subjects were recruited from the Neurological Department of the General Hospitals of Thessaloniki and Athens. The inclusion criteria were A) suffering from dementia, B) suffering from hallucinations according to the Neuropsychiatric Inventory (NPI), C) their caregivers were eager to cooperate. The study is in accordance with the ethical principles (Declaration of Helsinki) [12]. Their mean age was 75.83 years old (SD 8.76), and the mean years of education was 8.27 (SD 4.34). The criteria for the diagnosis of AD and MCI due to AD is in accordance with the National Institute of Neurological and Communicative Disorders and Stroke (NINCDS) and the Alzheimer’s Disease and Related Disorders Association (ADRDA). The patients were diagnosed with Alzheimer’s disease (AD), Vascular Dementia (VD), Lewy Body Dementia (DLB), Frontotemporal Dementia (FTD), Mild Cognitive Impairment (MCI), Mixed type (AD & VD) and Aids. The 48.3% of the sample (n = 29) were males. The demographic characteristics of the sample is shown on table 1.

### Procedure

This is a randomized, controlled, crossover trial. The NPI inventory applied to the family caregivers before any treatment and guidelines. The patients were randomly assigned to 6 different groups. The interventions lasted for 5 days and there were 2 days as a wash-out period. The interventions were applied every morning after breakfast. NPI questionnaire applied before any intervention, and on the morning of the 6^th^ day it was applied again, in order to evaluate the results of each intervention. The sequence of the treatments in each group was: Group 1: ABC, Group 2: ACB, Group 3: BAC, Group 4: BCA, Group 5: CAB and Group 6: CBA. Table 2 shows the sequence of the procedure.

### Interventions

The interventions used were: A) Validation Therapy (VT) in combination with psychoeducational program, B) Reminiscence Therapy (RT), and C) Music Therapy (MT). The inclusion criteria for the interventions were: A) to be pleasurable, B) have no side effects, C) are cost effective, and D) can be easily applied by informal caregivers.

### VT/psychoeducational program

VT focuses on accepting the reality of the PwD and includes techniques such as: Using non-threatening tone of voice, use simple words, maintain eye contact, and use touch when is necessary [13]. VT aims to alleviate negative feelings and enhance the positive ones. A psycho-educational program was conducted for the caregivers to be able to apply VT. Our psycho-educational program for the caregivers aimed to educate them about dementia, BPSD and other matters related to the disease. The program took place in face-to-face meetings and in online meetings. The program included 24 seminars, lasted for 12 weeks and the duration of each seminar was approximately 2 hours. The guideline given from the specialists was “when hallucinations occur, please change the subject and try to distract the patient by talking to him/ her for something else”.

### Reminiscence therapy (RT)

RT is used in order to recall past memorable events of the patient’s life. Photos, music, books, and letters can be used [14]. It involves the discussion of past experiences and events. The caregiver aims to involve the patient in a discussion and/or evaluation of the experiences [14]. In accordance with previous studies [15,16], the current trial used photo albums in order to recall past experiences. The intervention lasted 60 minutes per session and took place once a day after breakfast.

### Music therapy (MT)

The treatment belongs to the Stimulant Based Interventions [17]. It remains unknown which type of music has the most effective results [18]. The literature so far has no valid confirmation of how much time of MT is needed in order to achieve its best results [18]. In the current trial was used 45 minutes per session, five times a week, once per day every morning after breakfast. The caregiver applied the favorite music of each participant.

### Measures

#### Mini mental state examination (MSME)

MMSE is a 30-points questionnaire that is used to evaluate the cognitive status. It is used to estimate the severity of cognitive decline. The questionnaire examines registration, attention, recall, language, praxis, and orientation. Higher scores indicate better cognitive performance [19,20].

#### Addenbrooke’s cognitive examination revised (ACE-R)

ACE-R is a 100-point questionnaire that is used to evaluate the cognitive impairment. It includes MMSE. It is highly sensitive and can be used to support the diagnosis of dementia. Higher scores indicate better cognitive performance [21,22].

#### Geriatric scale of depression (GDS)

This scale is a questionnaire of 30 YES/NO questions that examines if the patient has depression. Higher scores indicate higher level of depression [23,24].

#### Functional rating scale for symptoms in dementia (FRSSD)

It is a scale to access the activities of daily living. The scale is a questionnaire which is completed by caregivers and includes 14 different daily activities, such as: eating, dressing, incontinence, speaking, sleeping, recognition of faces, personal hygiene, memory of names, memory of events, alertness, agitation, spatial orientation, emotional status, socialization. The scale is scored from 0–3 for each question (whereas 0 = fully independent and 3 = fully dependent). Higher scores indicate lower level of functionality (Activities of Daily Living) [25,26].

#### Neuropsychiatric inventody (NPI)

This questionnaire is given to the caregiver. It evaluates the frequency and severity of the symptoms, and the impact of each behaviour on the caregiver. The domain total score is the product of A) frequency multiplication severity score and B) the total score of the caregivers’ distress. A total score is obtained by summing all the domain total scores. It is a flexible, easy to use, and a valid and reliable tool. It evaluates a wide range of psychopathology, including hallucinations, and it can be used across different ethnic groups. In the current study only the sub questions that refer to wandering/aberrant motor behaviour were used [27,28].

### Data Analysis

Categorical variables were presented as percentages while continuous variables were presented as Mean value and Standard Deviation (SD). Wilcoxon signed-rank test was used because the distribution of the differences between the samples cannot be assumed to be normally distributed. Chi-square test was used to find differences in gender in the 6 groups, and finally, z value score was used in order to find the type of dementia in each group. P values less than 0.05 were considered statistically significant. SPSS 25.0 (IBM Inc., Armonk, NY) was used for the statistical analysis.

## RESULTS

The 67.2% of the patients were suffering from AD, 1.6% from VAD, 0.5% from DLB, 4.9% from PDD, 2.7% from FTD, 0.5% from Mixed dementia (AD and VAD), and 15.3% from Mild Cognitive Impairment (MCI). Percentages of the dementia type are shown on table 3.

According to Wilcoxon test the most effective non-pharmacological combination of the interventions for NPI Results was: VT/Psychoeducational program - MT- RT, which was applied in group 2. The baseline characteristics of group 2 are the following: The mean score of the MMSE test was 15.8 (SD 7.05), of the ACER test 47.9 (SD 21.57), of the GDS questionnaire 8.8 (SD 25.8), and of the FRSSD 6.25 (SD 8.70). Group 2 had AD patients (60%), MCI patients (10%), DLB patients (20%) and FTD patients (10%). Table 4 shows the characteristics of group 2.

Specifically, in group 2, VT/Psychoeducational program applied first and reduced the hallucinations effectively (p = 0.005). MT applied after VT/Psychoeducation program intervention and reduced the hallucinations (p = 0.007) and after that RT was applied, which reduced the hallucinations further (p = 0.022). Wilcoxon test was conducted in order to compare baseline NPI and final NPI. According to these results, it seems that there is no other effective combination that can reduce hallucinations in patients with dementia. Therefore, the most effective combination was found on group 2.

The abovementioned combination was also the most effective for the reduction of the caregivers’ burden. Specifically, in group 2: VT/Psychoeducational program was also applied in the first week and reduced the distress (p = 0.010). MT was applied in the second week and reduced the distress further (p = 0.023). RT was applied in the third week and reduced even caregivers’ burden (p = 0.036). Wilcoxon test was conducted in order to compare baseline NPI and final NPI. According to these results, it seems that there is no other effective combination that can reduce caregivers’ burden so much, because of the hallucinations of their patients. Therefore, the most effective combination for the caregivers’ distress was found on group 2, as well. The tables below show all the results (Table 5).

## DISCUSSION

Our study showed that the most effective combination of non-pharmacological interventions for the reduction of hallucinations in dementia and caregivers’ burden is: “Validation therapy/ Psycho-educational program followed by MT, followed by RT”. This is important because no trials so far have examined either alone or in combination of the non-pharmacological interventions for the treatment of hallucinations. To our knowledge, no trials, so far, have found an effective combination that can reduce hallucinations and caregivers’ burden. It is also critical to mention that no other combination of non-pharmacological interventions can reduce this unwanted behaviour. MT is a beneficial non-pharmacological intervention which is harmless. Some reviews have found positive evidence of MT on the reduction of a range of BPSD [29,30], but there is no evidence of MT effect on hallucinations. Furthermore, the literature so far, lacks evidence on the efficacy of RT on hallucinations.

In our study, we found that VT in combination with Psychoeducational program, when applied first, can reduce statistically significant the hallucinations in PwD and caregivers’ burden, as well. Caregivers who have not been educated, they may feel anxious and guilty, thinking that they should do something in order to reduce the hallucinations. However, because they are uneducated, they do not know how to react and therefore they feel more anxious. The Psychoeducational program helps the caregivers to understand better what hallucinations are and how they should be treated. This program, also, aimed to reduce the guilty feelings of the caregivers. According to our results the educational program helped the caregivers to reduce both the hallucinations and their distress.

However, some important questions need to be answered. If auditory hallucinations occur can MT be beneficial in that case? Our study support that MT can be beneficial. Which type of MT has the best results? In our study we used patients’ favorite music. The second question is: How long should the MT last? We used MT only for 5 days and we had significant results. The third question is: How long should RT last? We also used RT for one week and we had also beneficial results. The last question is: How long do the positive effects last? We have no results which can response this question because the design of the study did not include follow up of the patients.

Furthermore, according to our results, a sensory non-pharmacological intervention (MT), can reduce hallucinations further in contrast with a cognitive non-pharmacological intervention (RT). Therefore, a cognitive intervention in combination with a behavioral treatment is more effective than a cognitive intervention alone. Sensory stimulation intervention (MT) is more effective than a cognitive intervention (RT). Because of the complexity of the hallucinations, the caregivers need to be aware of a theoretical background first and then perform the other interventions. As for the PwD, it seems that if their caregivers know how to treat them better, then the hallucinations can be reduced.

The strength of the current study is that is a cross-over randomized designed trial, in order to avoid risk of bias. The sequence of the procedure does not interfere with the results. Therefore, the study has a strong methodology. In addition, apart from the heterogeneity of the sample, it seems that our results affect both genders, different types, and stages of dementia. The Psycho-educational program was very well-organized and detailed. NPI is a valid and trustworthy tool. Moreover, the study investigated an unexplored area and found some important results, which need further longitudinal research in a larger sample with the same interventions and follow up. It is also important to mention another interesting finding; the non-pharmacological intervention that reduced more the hallucinations in dementia is the same intervention that reduced the caregivers’ burden, as well. The caregivers’ burden is in dependence with the behaviour of the patient. On the other hand, the limitations of the current study are that each intervention lasted for only five days and there was no follow-up. However, this can be explained because of the efficient and rapid solutions that the caregivers asked for. Additionally, the interventions were administrated by the caregivers. Nevertheless, the psycho-educational program that guided the caregivers was strict and the psychologist was available for more explanations. Future studies should use large sample size, and RCT trials in order to confirm the useful role of the non-pharmacological interventions in the management of the hallucinations in dementia.

In sum, this study supports that there is a combination of non-pharmacological treatments that can be helpful for the reduction of the hallucinations in PwD and caregivers’ distress, too. Considering that the current pharmacological treatments have severe side-effects, the non-pharmacological interventions could be an effective alternative.

## Figures and Tables

**Table 1:** The demographic characteristics of the sample.

**Males, N (%)**	48.3% (N = 29)
**Age**	75.83 (8.76)
**Years of Education**	8.27 (4.34)
**MMSE**	17.47 (4.76)
**ACE-R**	49.55 (18.45)
**GDS**	9.27 (4.94)
**FRSSD**	19.62 (8.72)
**NPI Results**	7.57 (1.86)
**NPI Distress**	3.65 (0.86)

**Table 2:** The sequence of the procedure.

Group	Sequence	1^st^ Week	2^nd^ Week	3^rd^ Week
1	ABC	A	B	C
2	ACB	A	C	B
3	BAC	B	A	C
4	BCA	B	C	A
5	CAB	C	A	B
6	CBA	C	B	A

Intervention A= VT/ Psychoeducational program, Intervention B = RT and Intervention C = MT.

**Table 3:** Percentages of the dementia types.

AD	VAD	LBD	PDD	FTD	Mixed (AD & VAD)	MCI
67.2%	1.6%	0.5%	4.9%	2.7%	0.5%	15.3%

**Table 4:** Baseline characteristics of group 2.

MMSE	ACE-R	GDS	FRSSD	AD	MCI	DLB	FTD
15.8 (SD 7.05)	47.9 (SD 21.57)	8.8 (SD 25.8)	6.25 (SD 8.70)	60%	10%	20%	10%

**Table 5:** Results of the different order of interventions on Hallucinations and distress of caregivers.

**Group 1**	**Baseline NPI**	**A-B**	**B-C**	**NPI Baseline - NPI Final**
**Mean Score ± SD**	7 ± 1.95	6 ± 1.49 – 7 ± 1.95	7 ± 1.95 – 7 ± 1.76	7 ± 1.95 – 7 ± 1.76
**Percentiles**		6–8, 6–8.25	6–8.25, 5.50–8	6–8, 5.50–8
**p**		0.502	0.102	0.962
**Group 2**	**Baseline NPI**	**A-C**	**C-B**
**Mean Score ± SD**	8 ± 1.88	6 ± 1.79 – 5 ± 1.63	5 ± 1.63 – 4.5 ± 1.60
**Percentiles**		4–8, 4–6	4–6, 3.25–6
**p**		0.007	0.022
**Group 3**	**Baseline NPI**	**B-A**	**A-C**	**NPI Baseline - NPI Final**
**Mean Score ± SD**	8 ± 2.22	8 ± 2.22 – 5 ± 1.83	5 ± 1.83 – 7 ± 2.06	8 ± 2.22 – 7 ± 2.06
**Percentiles**		6–9, 4–8	4–8, 4–8.25	6–9, 4–8.25
**p**		0.017	0.244	0.224
**Group 4**	**Baseline NPI**	**B-C**	**C-A**	**NPI Baseline - NPI Final**
**Mean Score ± SD**	8 ± 1.85	8 ± 1.85 – 7 ± 1.63	7 ± 1.63 – 6 ± 1.52	8 ± 1.85 – 6 ± 1.52
**Percentiles**		6–9, 6–8.25	6–8.25, 5.50–6.50	6–9, 5.50–6.50
**p**		0.102	0.151	0.041
**Group 5**	**Baseline NPI**	**C-A**	**A-B**	**NPI Baseline - NPI Final**
**Mean Score ± SD**	7 ± 1.63	7 ± 2.01 – 6 ± 1.57	6 ± 1.57 – 6 ± 1.49	7 ± 1.63 – 6 ± 1.49
**Percentiles**		4–8.25, 4–6.50	4–6.50, 6–8	6–8.25, 6–8
**p**		0.036	0.616	0.847
**Group 6**	**Baseline NPI**	**C-B**	**B-A**	**NPI Baseline - NPI Final**
**Mean Score ± SD**	8.5 ± 2.22	8 ± 1.25 – 8.5 ± 2.22	8 ± 1.25 – 8.5 ± 2.22	8.5 ± 2.22 – 7 ± 1.95
**Percentiles**		6–9, 6–9.25	6–9, 6–9.25	6–9.25, 4–8
**p**		0.517	0.517	0.037
**Group 1**	**Baseline NPI**	**A-B**	**B-C**	**NPI Baseline - NPI Final**
**Mean Score ± SD**	3 ± 0.51	3 ± 0.63 – 3 ± 0.51	3 ± 0.51 – 3 ± 0.48	3 ± 0.51 – 3 ± 0.48
**Percentiles**		2–3, 3–4	3–4, 3–4	3–4, 3–4
**p**		0.123	0.317	1
**Group 2**	**Baseline NPI**	**A-C**	**C-B**
**Mean Score ± SD**	3.8 ± 0.91	3.5 ± 0.91 – 3 ± 0.96	3 ± 0.96 – 2 ± 0.69
**Percentiles**		3–5, 3–5	3–5, 2–4
**p**		0.023	0.036
**Group 3**	**Baseline NPI**	**B-A**	**A-C**	**NPI Baseline - NPI Final**
**Mean Score ± SD**	3 ± 0.94	3 ± 0.94 – 3 ± 0.63	3 ± 0.63 – 3 ± 0.99	3 ± 0.94 – 3 ± 0.99
**Percentiles**		2.75–4, 2–3	2–3, 2–3.25	2.75–4, 2–3.25
**p**		0.102	0.841	1
**Group 4**	**Baseline NPI**	**B-C**	**C-A**	**NPI Baseline - NPI Final**
**Mean Score ± SD**	4 ± 1.03	4 ± 1.03 – 3.5 ± 0.96	3.5 ± 0.96 – 3 ± 0.63	4 ± 1.03 – 3 ± 0.63
**Percentiles**		3–5, 3–4.25	3–4.25, 2–3	3–5, 2–3
**p**		0.157	0.123	0.052
**Group 5**	**Baseline NPI**	**C-A**	**A-B**	**NPI Baseline - NPI Final**
**Mean Score ± SD**	3 ± 0.63	3 ± 0.56 – 3 ± 0.56	3 ± 0.56 – 3 ± 0.63	3 ± 0.63 – 3 ± 0.63
**Percentiles**		3–3.25, 2.75–3	2.75–3, 3–4	3–4, 3–4
**p**		1	0.328	1
**Group 6**	**Baseline NPI**	**C-B**	**B-A**	**NPI Baseline - NPI Final**
**Mean Score ± SD**	4 ± 0.73	4 ± 0.73 – 4 ± 0.66	4 ± 0.66 – 3 ± 0.67	4 ± 0.73 – 3 ± 0.67
**Percentiles**		3–4, 3.75–4.25	3.75–4.25, 3–4	3.75–5, 3–4
**p**		0.317	0.059	0.062

## ABBREVIATIONS

AD: Alzheimer’s Disease
BPSD: Behavioural and Psychological Symptoms in Dementia
DLB: Lewy Body Dementia
FTD: Frontotemporal Dementia
MCI: Mild Cognitive Impairment
MT: Music Therapy
PDD: Parkinson’s Dementia
PwD: Patients with Dementia
RT: Reminiscence Therapy
VaD: Vascular Dementia
VT: Validation Therapy

## References

[1] Lin W, Xie YC, Cheng PY (2018) Association of visual hallucinations with very mild degenerative dementia due to dementia with Lewy bodies. Plos One 13(10): e0205909. 30321234 10.1371/journal.pone.0205909 PMC6188892

[2] Ukai K (2019) Tactile hallucinations in dementia with lewy bodies. Psychogeriatrics 19(5): 435–439. 30734409 10.1111/psyg.12407

[3] Tsunoda N, Hashimoto M, Ishikawa T (2018) Clinical features of auditory hallucinations in patients with dementia with lewy bodies: A soundtrack of visual hallucinations. The Journal of Clinical Psychiatry 79(3): 17m11623. 10.4088/JCP.17m11623 29742332

[4] Swann P and O’Brien JT (2019) Management of visual hallucinations in dementia and parkinson’s disease. International Psychogeriatrics 31(6): 815–836. 30398127 10.1017/S1041610218001400

[5] Tartaglia MC, Kertesz A, Ang LC (2008) Delusions and hallucinations in frontotemporal dementia: A clinicopathologic case report. Cognitive and Behavioral Neurology 21(2): 107–110. 18541988 10.1097/WNN.0b013e3181799e19

[6] Asahara Y, Mukai T, Suda M (2021) Etiology and treatment approach for visual hallucinations in PD dementia. In Dementia in Parkinson’s Disease. IntechOpen.

[7] Small GW (2020) Managing the burden of dementia related delusions and hallucinations. The Journal of Family Practice 69(7 Suppl): S39–S44. 33104106

[8] Hershey LA and Coleman-Jackson R (2019) Pharmacological management of dementia with lewy bodies. Drugs & Aging 36(4): 309–319. 30680679 10.1007/s40266-018-00636-7 PMC6435621

[9] Powell A, Matar E, Lewis SJ (2020) Treating hallucinations in Parkinson’s disease. Expert Review of Neurotherapeutics: 1–14. 10.1080/14737175.2021.1851198 33183105

[10] Cummings J, Ballard C, Tariot P (2018) Pimavanserin: Potential treatment for dementia-related psychosis. The Journal of Prevention of Alzheimer’s Disease 5(4): 253–258. 10.14283/jpad.2018.29 PMC6413822 30298184

[11] Dyer SM, Harrison SL, Laver K (2018) An overview of systematic reviews of pharmacological and non-pharmacological interventions for the treatment of behavioral and psychological symptoms of dementia. International Psychogeriatrics 30(3): 295–309. 29143695 10.1017/S1041610217002344

[12] World Medical Association (2013) World medical association declaration of helsinki: Ethical principles for medical research involving human subjects. JAMA 310(20): 2191–2194. 24141714 10.1001/jama.2013.281053

[13] Cai Y, Li L, Xu C (2020) The effectiveness of non-pharmacological interventions on apathy in patients with dementia: A systematic review of systematic reviews. Worldviews on Evidence-Based Nursing 17(4): 311–318. 32767834 10.1111/wvn.12459

[14] Park K, Lee S, Yang J (2019) A systematic review and meta-analysis on the effect of reminiscence therapy for people with dementia. International Psychogeriatrics 31(11): 1581–1597. 30712519 10.1017/S1041610218002168

[15] Redulla R (2020) Reminiscence therapy for dementia. Issues in Mental Health Nursing 41(3): 265–266. 31714840 10.1080/01612840.2019.1654572

[16] Lök N, Bademli K, Selçuk-Tosun A (2019) The effect of reminiscence therapy on cognitive functions, depression, and quality of life in alzheimer patients: Randomized controlled trial. International Journal of Geriatric Psychiatry 34(1): 47–53. 30246408 10.1002/gps.4980

[17] Moreno-Morales C, Calero R, Moreno-Morales P (2020) Music therapy in the treatment of dementia: A systematic review and meta-analysis. Frontiers in Medicine 7: 160. 32509790 10.3389/fmed.2020.00160 PMC7248378

[18] Ueda T, Suzukamo Y, Sato M (2013) Effects of music therapy on behavioral and psychological symptoms of dementia: A systematic review and meta-analysis. Ageing Research Reviews 12(2): 628–641. 23511664 10.1016/j.arr.2013.02.003

[19] Folstein MF, Robins LN, Helzer JE (1983) The mini-mental state examination. Archives of General Psychiatry 40(7): 812–822. 6860082 10.1001/archpsyc.1983.01790060110016

[20] Fountoulakis KN, Tsolaki M, Chantzi H (2000) Mini mental state examination (MMSE): A validation study in Greece. American Journal of Alzheimer’s Disease & Other Dementias® 15(6): 342–345.

[21] Mathuranath PS, Nestor PJ, Berrios GE (2000) A brief cognitive test battery to differentiate Alzheimer’s disease and frontotemporal dementia. Neurology 55(11): 1613–1620. 11113213 10.1212/01.wnl.0000434309.85312.19

[22] Konstantinopoulou E, Kosmidis MH, Ioannidis P (2011) Adaptation of addenbrooke’s cognitive examination-revised for the Greek population. European Journal of Neurology 18(3): 442–447. 20649904 10.1111/j.1468-1331.2010.03173.x

[23] Yesavage JA, Brink TL, Rose TL (1982) Development and validation of a geriatric depression screening scale: A preliminary report. Journal of Psychiatric Research 17(1): 37–49. 7183759 10.1016/0022-3956(82)90033-4

[24] Fountoulakis KN, Tsolaki M, Iacovides A (1999) The validation of the short form of the geriatric depression scale (GDS) in Greece. Aging Clinical and Experimental Research 11(6): 367–372. 10738851 10.1007/BF03339814

[25] Hutton JT, Dippel RL, Loewenson RB (1988) Functional rating scale for the symptoms of dementia. Handbook of Geriatric Assessment: 77–80.

[26] Tsolaki M (1997) Neuropsychological evaluation of the elderly. Melissa, Thessaloniki.

[27] Cummings JL, Mega M, Gray K (1994) The neuropsychiatric inventory: Comprehensive assessment of psychopathology in dementia. Neurology 44(12): 2308–2314. 7991117 10.1212/wnl.44.12.2308

[28] Politis AM, Mayer LS, Passa M (2004) Validity and reliability of the newly translated Hellenic Neuropsychiatric Inventory (H-NPI) applied to Greek outpatients with alzheimer’s disease: A study of disturbing behaviors among referrals to a memory clinic. International Journal of Geriatric Psychiatry 19(3): 203–208. 15027034 10.1002/gps.1045

[29] Lam HL, Li WTV, Laher I (2020) Effects of music therapy on patients with dementia - A systematic review. Geriatrics 5(4): 62. 10.3390/geriatrics5040062 PMC7709645 32992767

[30] Ray KD and Götell E (2018) The use of music and music therapy in ameliorating depression symptoms and improving well-being in nursing home residents with dementia. Frontiers in Medicine 5: 287. 30356835 10.3389/fmed.2018.00287 PMC6190855

